# Nanoplastics causes heart aging/myocardial cell senescence through the Ca^2+^/mtDNA/cGAS-STING signaling cascade

**DOI:** 10.1186/s12951-024-02375-x

**Published:** 2024-03-06

**Authors:** Kaihao Wang, Yipeng Du, Peixin Li, Chang Guan, Min Zhou, Lanlan Wu, Zengfu Liu, Zheng Huang

**Affiliations:** Department of Cardiology, The First Affiliated Hospital of Guangzhou Medical University, Guangzhou, China

**Keywords:** Nanoplastics, Cardiomyocytes, Aging, Mitochondria, Inflammation

## Abstract

**Background:**

Nanoplastics (NPs) are now a new class of pollutants widely present in the soil, atmosphere, freshwater and marine environments. Nanoplastics can rapidly penetrate cell membranes and accumulate in human tissues and organs, thus posing a potential threat to human health. The heart is the main power source of the body. But up to now, the toxicological effects of long-term exposure to nanoplastics on the heart has not been revealed yet.

**Results:**

We evaluated the effects of long term exposure of nanoplastics on cardiac cell/tissue in vitro and in vivo model. Furthermore, we explored the molecular mechanism by which nanoplastics exposure causes myocardial cell senescence. Immunohistochemistry, indirect immunofluorescence and ELISA were performed to detect the effects of nanoplastics on heart aging. We found that nanoplastics were able to induce significant cardiac aging through a series of biochemical assays in vivo. In vitro, the effects of nanoplastics on cardiac cell were investigated, and found that nanoplastics were able to internalize into cardiomyocytes in time and dose-dependant manner. Further biochemical analysis showed that nanoplastics induces cardiomyocytes senescence by detecting a series of senescence marker molecules. Molecular mechanism research shows that nanoplastics may cause mitochondrial destabilization by inducing oxidative stress, which leads to the leakage of mtDNA from mitochondria into the cytoplasm, and then cytoplasm-localized mt-DNA activates the cGAS-STING signaling pathway and promotes inflammation response, ultimately inducing cardiomyocytes senescence.

**Conclusions:**

In this work, we found that nanoplastics exposure induces premature aging of heart. Current research also reveals the molecular mechanism by which nanoplastics induces cardiomyocyte senescence. This study laid the foundation for further studying the potential harm of nanoplastics exposure on heart.

**Graphical abstract:**

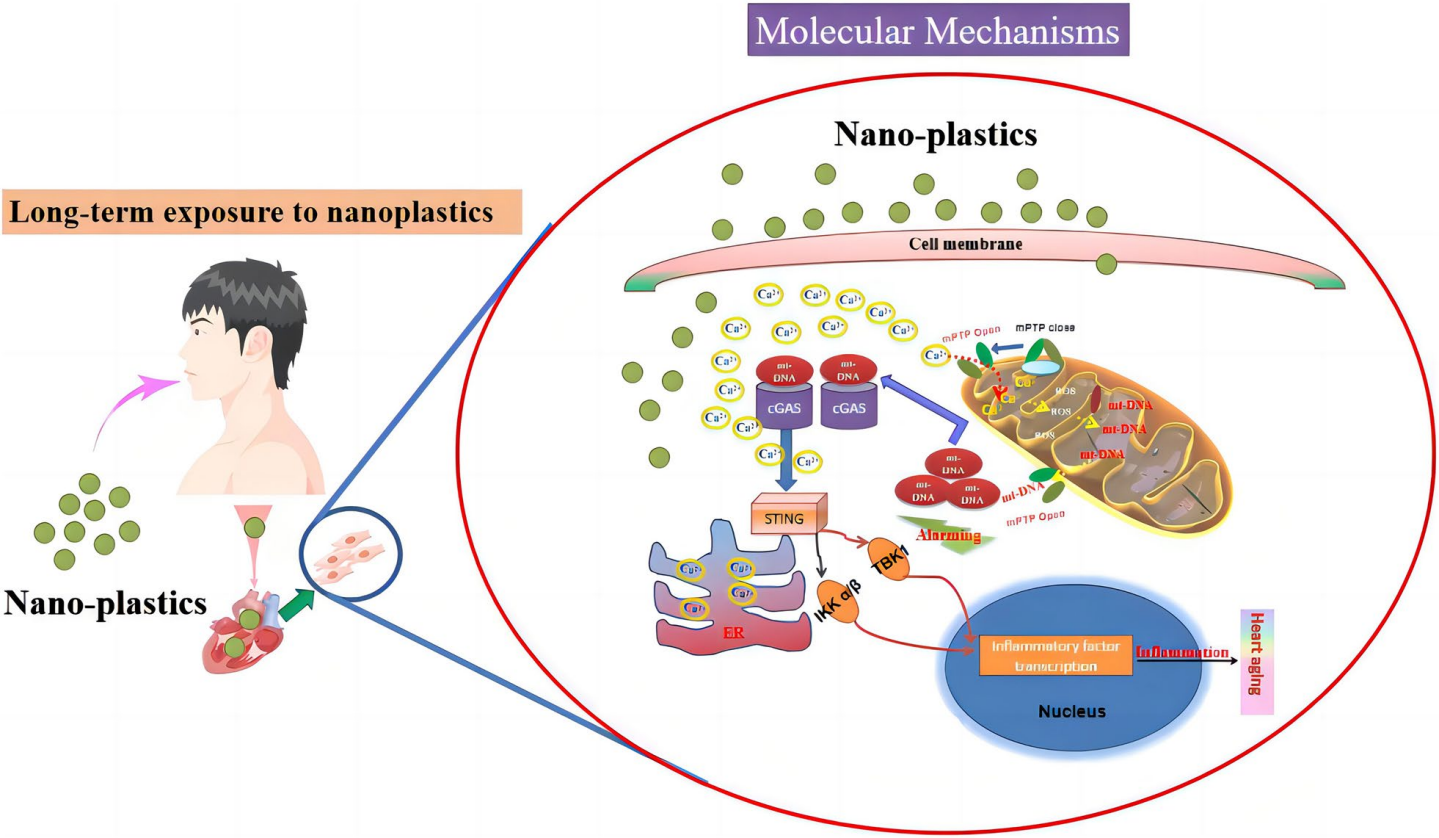

**Supplementary Information:**

The online version contains supplementary material available at 10.1186/s12951-024-02375-x.

## Background

Plastics are used in a wide range of applications in industry, communications and medicine [1]. Since the 1950s, the consumption of plastics has seen a rapid increase. The massive production and use of plastics, coupled with inadequate recycling measures, has led to a large amount of plastics entering the environment, which in turn has caused serious environmental pollution [2]. After entering the environment, plastic fragments are gradually formed into microscopic plastic particles by various physical, chemical and microbial effects [3]. The plastic particles in the size range from 1 μm to 5 mm are defined “microplastics”, and size (1–1000 nm) is defined as nanoplastics [4]. Microplastics pose a great threat to the ecological environment because they are widespread around the world, difficult to degrade and difficult to remove effectively [4]. Plastics can enter the natural environment through a variety of pathways, resulting in the widespread presence of microplastic particles in the environment [5]. The wide distribution, small particle size, and hydrophobicity of nanoplastics make them easy to ingest by organisms and transmit through the food chain, ultimately causing adverse effects on ecosystems [6]. In recent years, nano/microplastic pollution has attracted widespread attention from scientists worldwide and has become a hot spot in environmental protection and pollution prevention and control.

The biological toxicity aspects of nanoplastics have received extensive attention. Current toxicological studies on plastics are mainly focused on digestive metabolism, reproductive toxicity and neurotoxicity [7]. Studies have shown that nanoplastics can enter the esophagus, stomach and intestine through the human oral cavity, causing disruption of the intestinal flora [8]. In addition, nanoplastics have adverse effects on embryonic and offspring development [9]. Furthermore, nanoplastics can induce widespread neurotoxicity, leading to behavioral abnormalities and depression in the experimental animals [10].

Microplastics enter the body and can be distributed in the blood and organs, persistent microplastic exposure causes damage to the corresponding tissues and organs [11]. Studies have reported damage to kidney cell and tissue caused by nanoplastics [12–14]. The heart is one of the most important organs in the body, and its main function is to provide power for blood flow [15]. However, to date, the effects of long-term exposure to nanoplastics on heart damage have not been revealed.

Aging of the heart is a common risk factor for many diseases. Here, we investigated the effect of nanoplastics on cardiac aging. We found that nanoplastics caused heart premature aging, inflammation and oxidative stress. Furthermore, in vitro, the underlying molecular mechanisms of nanoplastics causing cardiomyocytes senescence were revealed: nanoplastics treatment led to mitochondrial oxidative stress, which in turn led to leakage of mtDNA into the cytoplasm, thereby activating the cGAS-STING signaling pathway, ultimately causing the inflammation and senescence of cardiomyocytes. Taken together, this study shows that long-term exposure to nanoplastics contributes to aging and inflammatory damage in the heart.

## Results

### Nanoplastics (NPs) enrichment in mouse heart tissue in vivo

**Fig. 1:**
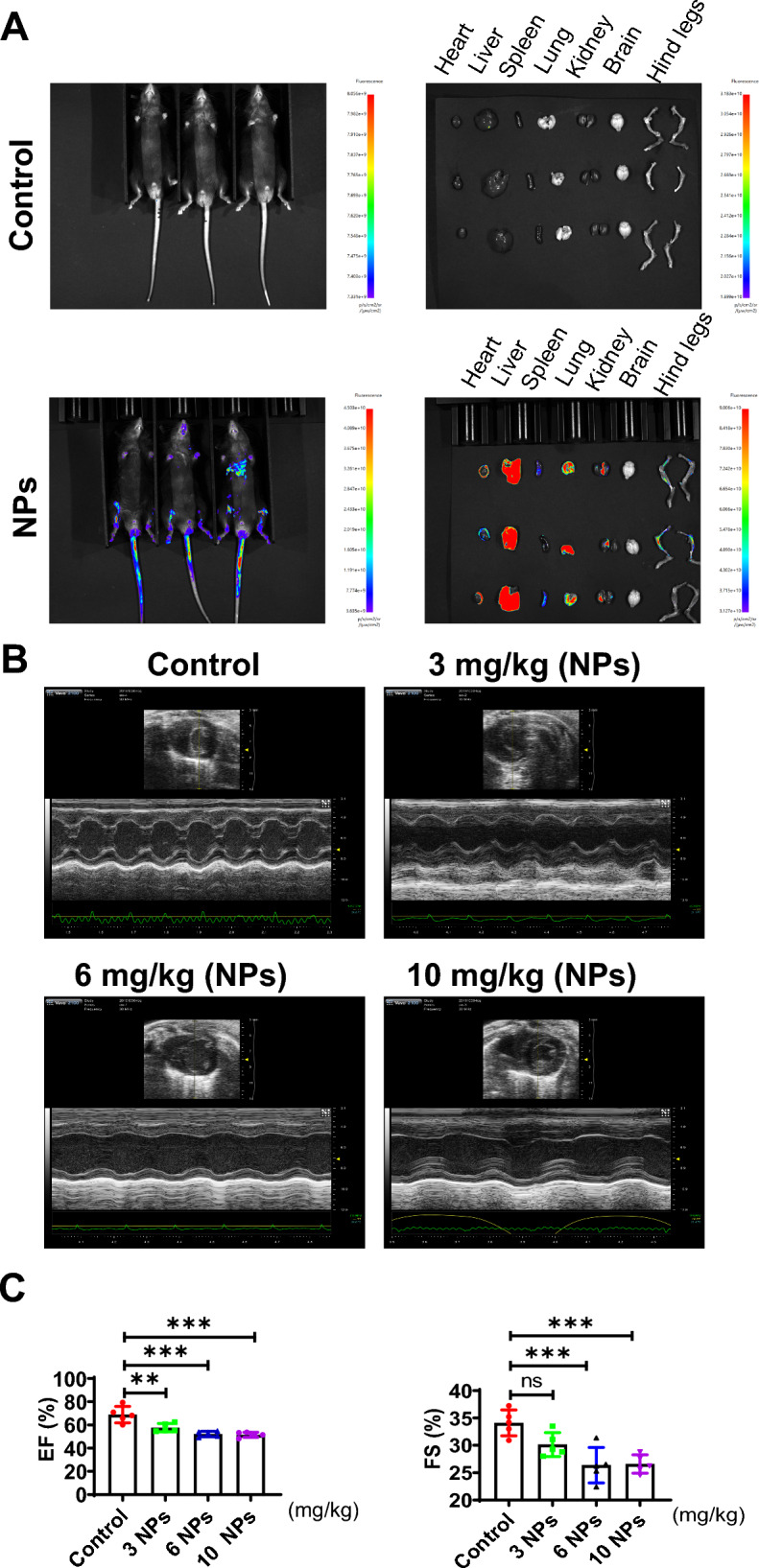
Distribution of NPs in organs in the body. **A** NPs are enriched in all major organs in the body. NPs were injected from the tail vein for 60 min, and animals were observed using a small animal live imaging system (Tanon, Shanghai, China). **B** The effect of NPs on cardiac tissue was detected by Doppler color ultrasound. **C** Effect of NPs on ejection fraction. An asterisk indicates a significant interference

We first investigated the in vivo distribution and localization of nanoplastics (NPs) by injection of fluorescently labeled nanoplastics through the tail vein. NPs were localized and enriched in the myocardial tissue of mice (Fig. 1A). In addition, NPs were also localized in other organs, including the liver and kidney, indicating that the in vivo distribution of NPs was widespread.

After NPs treatment for 8 weeks, we observed the effect of NPs on heart tissue using Doppler color ultrasound. The results revealed that NPs had potential effects on cardiac tissue (Fig. 1B). Furthermore, we evaluated the effects of NPs on cardiac contractile function and found that NPs treatment down-regulated EF (Ejection fraction) and FS (Fractional shortening), but NPs did not cause changes in cardiac function, EF and FS were still within the normal physiological range (the physiological range of EF: 55–65%; the physiological range of FS: 25–35%) (Fig. 1C).

### Nanoplastics caused damage to myocardial tissue

**Fig. 2:**
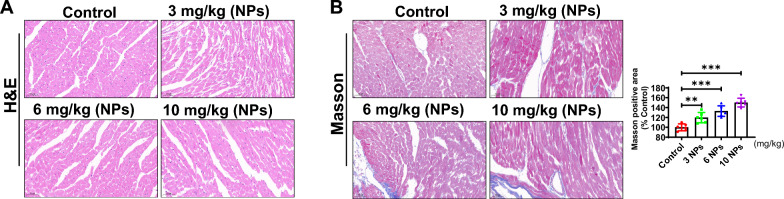
Effect of NPs on myocardial tissue damage. **A** Assessment of the effect of NPs on cardiac tissue by HE staining. **B** Effect of NPs on cardiac fibrosis by Masson staining. The same letter within column represents no significant difference (p > 0.05), different letters within column indicate significant difference (p < 0.05)

To assess the effects of NPs on pathological changes in the mouse myocardium, the experimental mice were treated with nano-scaled microplastics. After 8 weeks of NPs treatment, myocardial tissues were sampled, and HE staining results showed that myocardial cells in the control group were neatly arranged with normal structure, and no obvious inflammatory cell infiltration was seen (Fig. 2A). However, in the NPs-treated group, the cells were disordered and inflammatory cell infiltration was seen, indicating that NPs caused inflammatory damage to the myocardial tissue. Furthermore, Masson staining showed that NPs caused fibrosis in the heart tissue compared with the control group (Fig. 2B).

### Nanoplastics induced aging in mouse heart

**Fig. 3:**
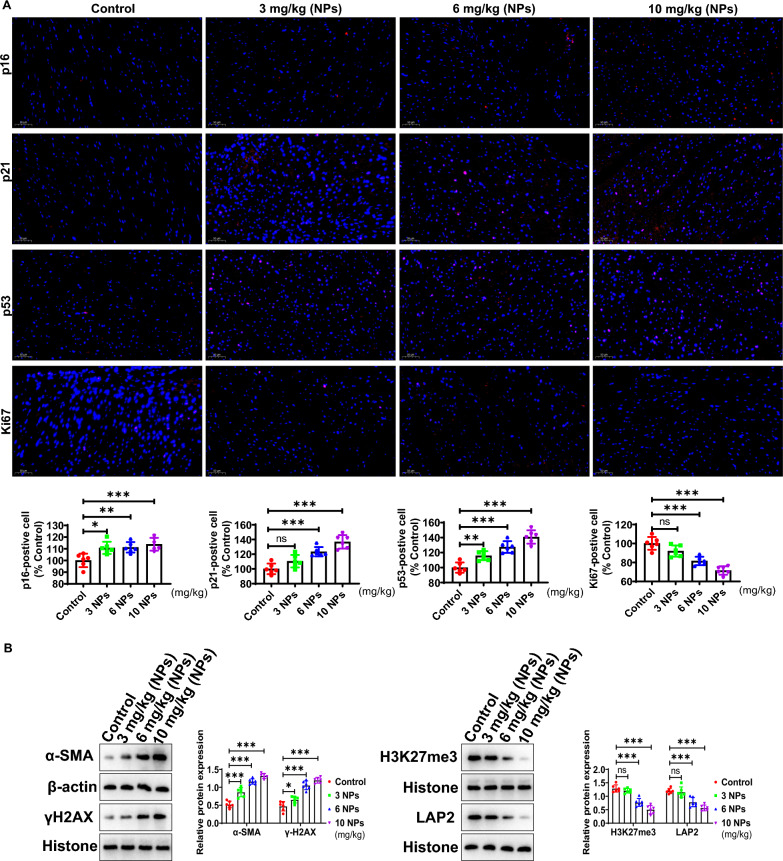
Effect of NPs on the aging of cardiac tissues. **A** Detection of p16/p21/p53 expression by immunohistochemistry. **B** Analysis of the expression of α-SMA, H3K27me3, γH2AX and LAP2 expression. Different letters indicate significant differences (p < 0.05)

The effects of NPs on the expression of the aging marker molecules (p16, p21 and p53) were investigated by immunohistochemistry. The results showed that the expression levels of senescence markers (p16, p21 and p53) were obviously increased (Fig. 3A). However, the expression of Ki67, a marker of cell proliferation, was clearly decreased (Fig. 3A). Figure 3B shows that the expression of α-SMA, a marker of fibrosis, was apparently elevated after NPs treatment. Furthermore, Western-blot analysis showed that H3K27me3 (heterochromatin marker) expression was obviously reduced. However, the protein expression of γH2AX (DNA damage marker) was significantly enhanced. In addition, the expression of LAP2 was also obviously reduced in the nanoplastics-treated group (Fig. 3B). Taken together, the above findings suggest that NPs caused premature aging of myocardial tissue.

### Effect of nanoplastics on inflammation level in mouse heart tissue

**Fig. 4:**
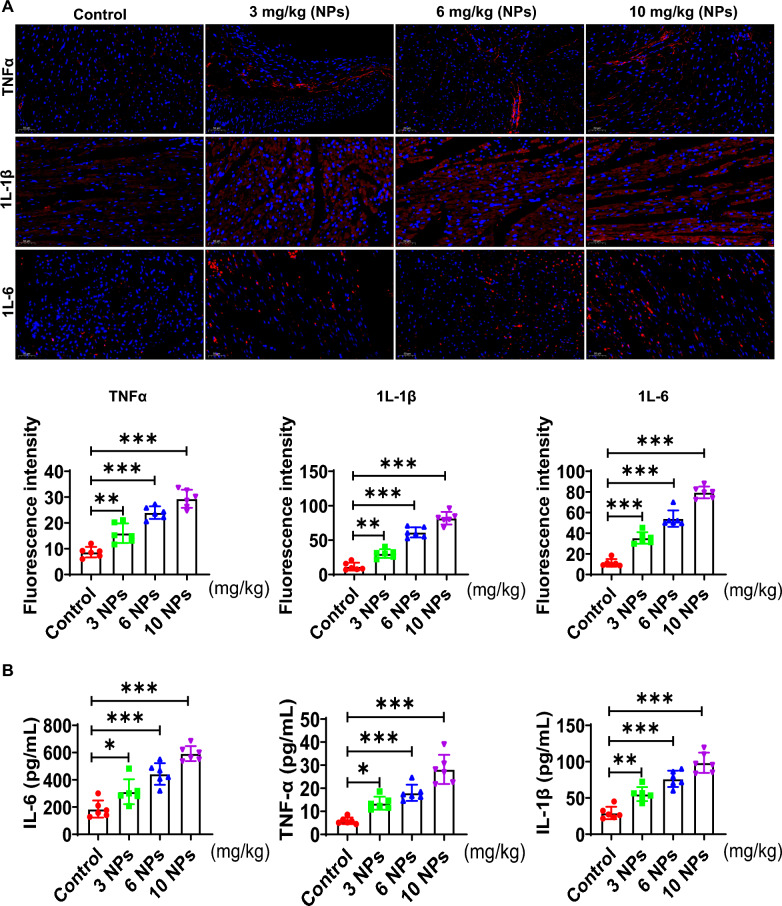
Effect of NPs on inflammation in mouse heart tissue. **A** Detection of TNFα, IL-1β, and IL-6 expression by IHC. **B** Effect of nano-scaled microplastics on pro-inflammatory factor level detected by ELISA. Different letters indicate significant differences (p < 0.05)

In this part, the effect of NPs on the inflammation of cardiac tissue was evaluated. First, immunohistochemical results showed that the expression levels of IL-6, TNFα and IL-1β were increased in the nano-scaled microplastic-treated group (Fig. 4A). Furthermore, the expression of inflammatory factors in myocardial tissues was detected by ELISA, and results showed that IL-6 and TNFα and IL-1β (pro-inflammatory factors) were apparently increased (Fig. 4B). These observations suggest that NPs caused significant inflammatory damage in myocardial tissues.

### Effect of nanoplastics on oxidative stress in mouse heart tissue

**Fig. 5:**
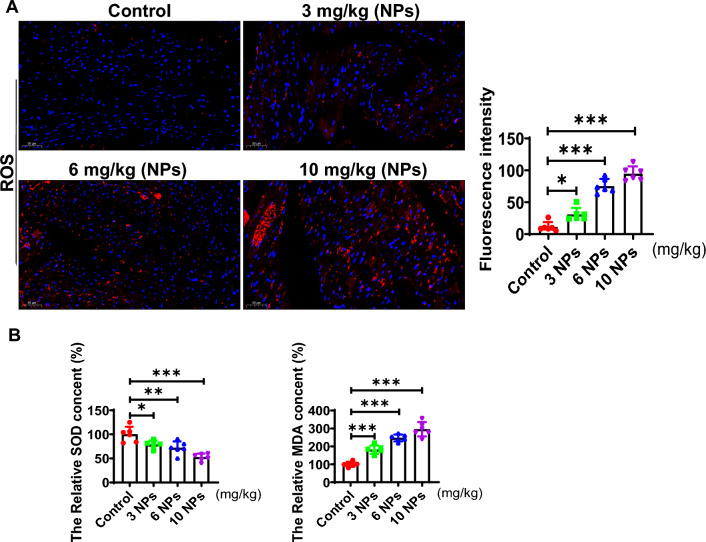
NPs treatment elevated ROS levels in myocardial tissues. **A** Effect of NPs on ROS expression levels. **B** Effect of NPs on MDA and ROS levels. The same letter within column represents no significant difference (p > 0.05), different letters within column indicate significant difference (p < 0.05)

We explored the effect of NPs on ROS levels by DHE, and the results showed that the ROS level was elevated in the nanoplastics-treated group (Red fluorescent signal) (Fig. 5A). Moreover, the expression level of SOD was noticeably reduced, and the level of MDA (malonaldehyde) was increased (Fig. 5B). These results suggest that NPs caused oxidative stress in the myocardial tissue.

### Nanoplastics were internalized into cardiomyocytes

**Fig. 6:**
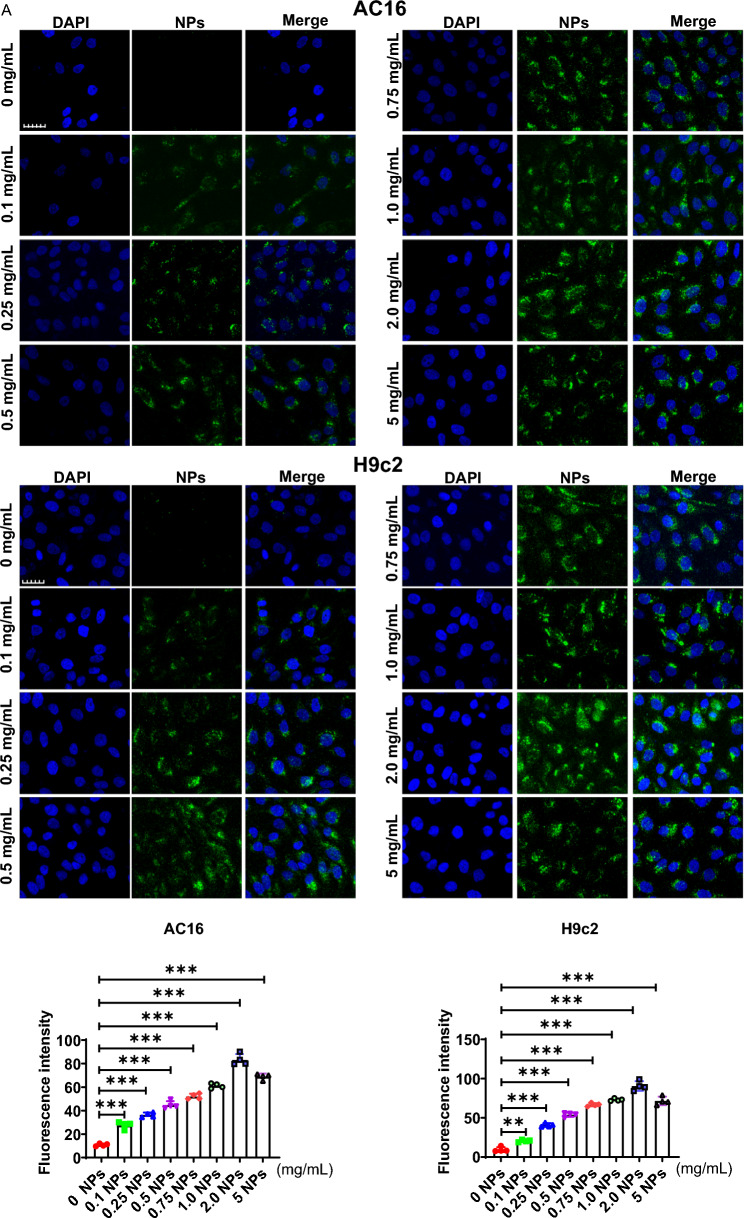

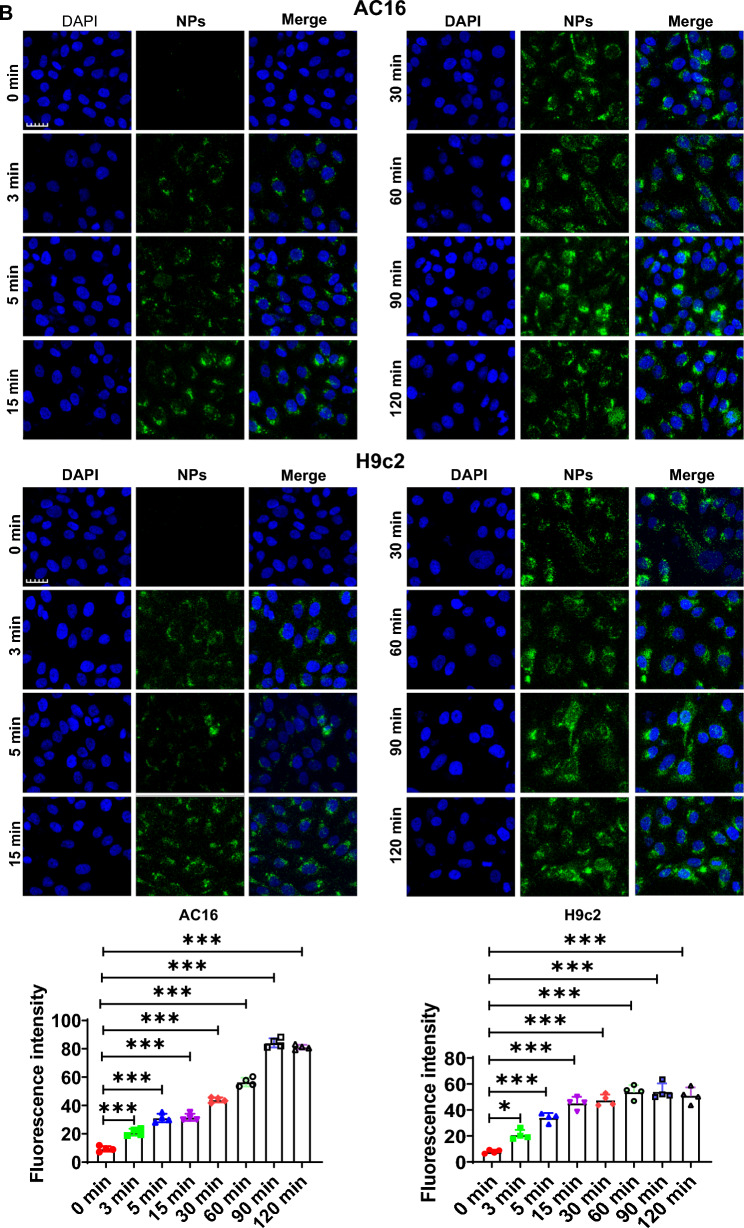
Internalization kinetics of NPs. **A** Internalization of NPs into cardiomyocytes cells in a dose-dependent manner. Cardiomyocytes were treated with NPs at 0–5.0 mg/mL. **B** Internalization of NPs into cardiomyocytes in a time-dependent manner. Cardiomyocytes were treated with NPs for 0–120 min. The samples were analyzed under the laser scanning confocal microscopy. Fluorescence images were analyzed and processed with FluoView FV3000 software (Olympus). The same letter within column represents no significant difference (p > 0.05); different letters within column indicate significant difference (p < 0.05)

In this part, we mainly assessed whether NPs could be internalized into cardiomyocytes (H9c2 and AC16). First, dose-dependent experiments were performed in which myocardial H9c2 cells were incubated with different concentrations of NPs (0, 0.1, 0.25, 0.5, 0.75, 1.0, 2.0 and 5.0 mg/mL) for 60 min, and found that NPs were able to internalize into the cells in a dose-dependent manner (Fig. 6A). On this basis, we performed time-dependent experiments. Based on the dose-dependent results, cells were incubated with 0.25 mg/mL of NPs for different time points (0–120 min). Confocal microscope observation showed that the fluorescently labeled NPs were able to internalize into the cardiomyocytes and exhibited the maximum fluorescence signal at 60 min (Fig. 6B). Taken together, these results suggest that NPs are able to internalize into cells with a time- and dose-dependent pattern.

### Nanoplastics stimulation causes cardiomyocytes senescence

**Fig. 7:**
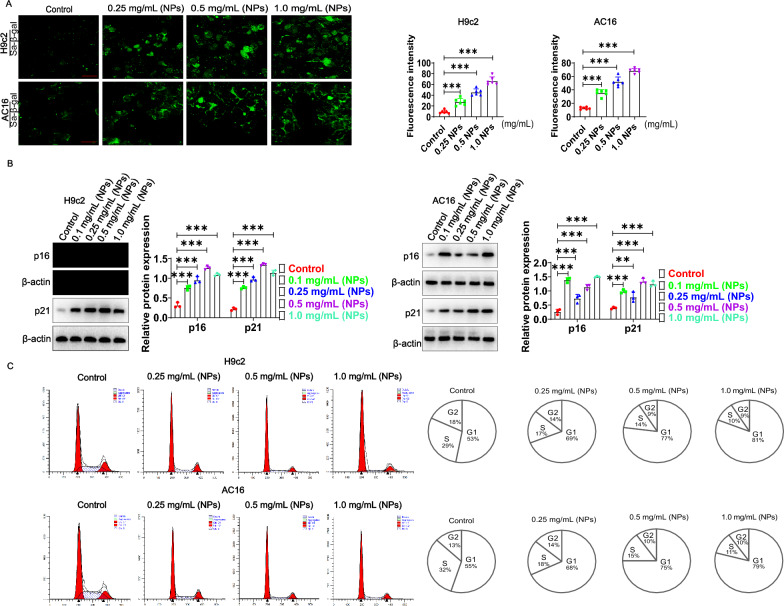
Effect of NPs on cardiomyocytes senescence. **A** Effect of NPs on Sa-β-gal expression. Different concentration of NPs was used to treat cells for 60 min. **B** Assessment of the effect of NPs on p16 and p21. **C** Effect of NPs on the cell cycle. Different letters within column indicate significant difference (p < 0.05)

The effect of NPs on cardiomyocyte senescence was evaluated in vitro experiments. For this purpose, two cell models (H9c2 and AC16) were used. First, one of the classical markers of senescence (Sa-β-gal) was detected using the CellEvent™ Senescence Green Detection Kit (Cat No: C10850, purchased from Invitrogen). The results showed that the percentage of Sa-β-gal-positive cells was significantly increased in the two cardiomyocyte models (Fig. 7A). Furthermore, the expression of p16 and p21 was also assessed by Western-blot, which showed a significant increase in the expression of p16 and p21 (Fig. 7B). These findings indicated that cardiomyocytes underwent significant senescence. Further work illustrated that the cell cycle was also inhibited after NPs treatment (Fig. 7C). Based on the above findings, we selected 0.2 mg/mL of NPs for the following experiments.

### Exploring the potential molecular mechanisms of cardiomyocyte senescence caused by nanoplastics

**Fig. 8:**
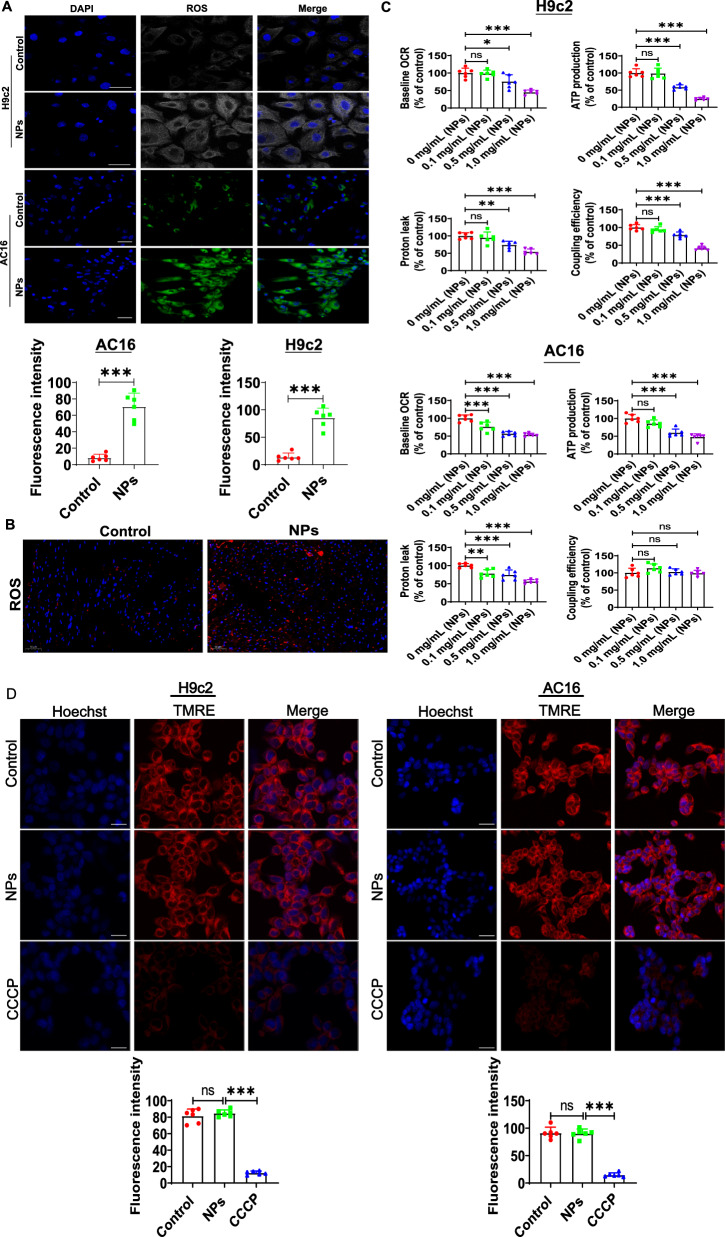

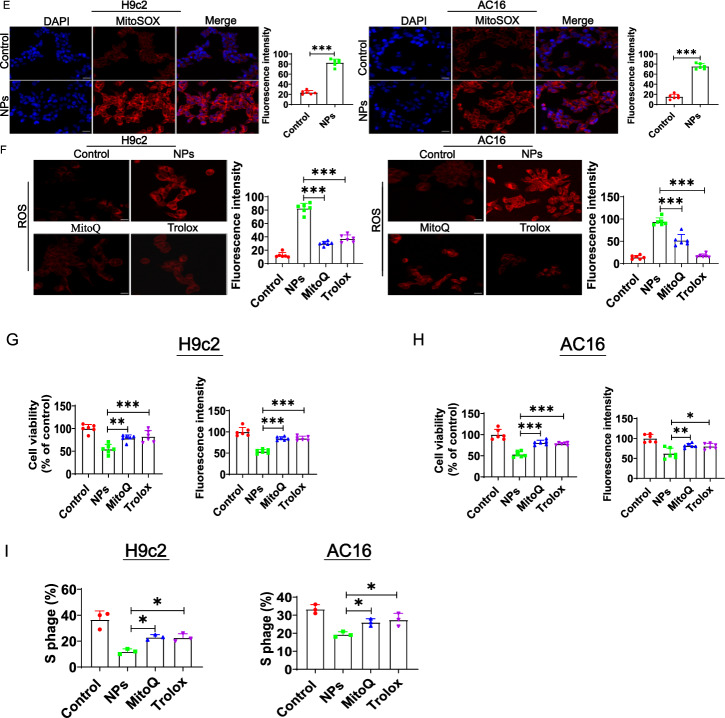

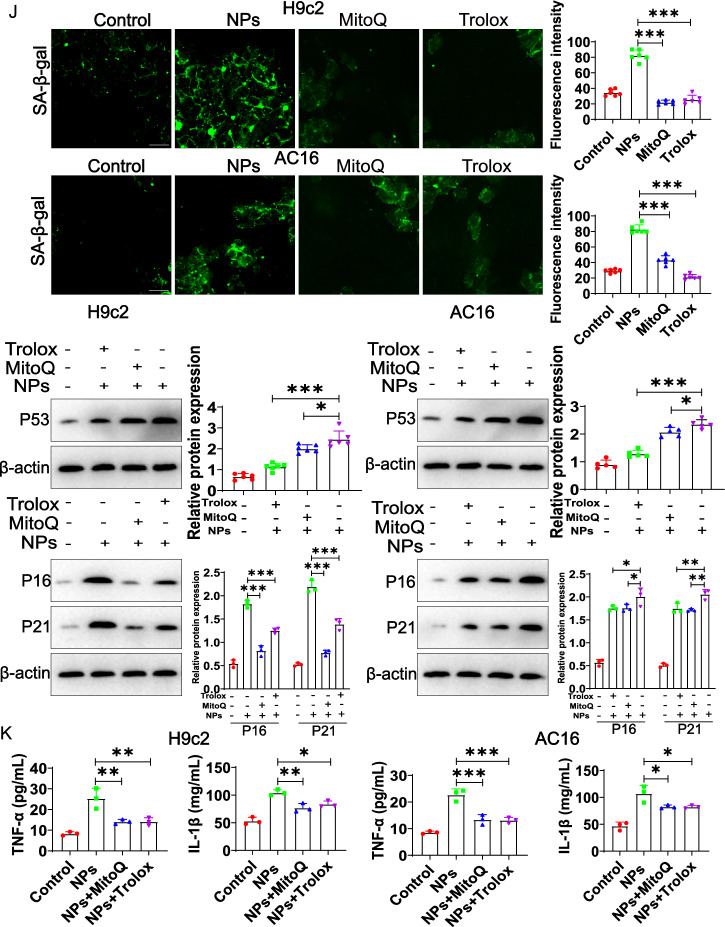
Effect of NPs on oxidative stress. **A** Effect of NPs on ROS expression in H9c2 and AC16 cell models. **B** Effect of NPs on ROS in vivo. **C** Effect of NPs on oxidative phosphorylation status. **D** Effect of NPs on the permeability of mitochondrial membranes. **E** Effect of NPs on redox reactions. **F** Removal of ROS by MitoQ and Trolox. **G** MitoQ and Trolox increased cell survival. **H** MitoQ and Trolox reduced cellular senescence. **I** MitoQ and Trolox improved the cell cycle. **J** Trolox and MitoQ blocked cellular senescence. **K** Trolox and MitoQ reduced the expression of inflammatory factors. An asterisk indicates a significant interference

Oxidative stress is an important factor in aging, and many studies have reported that NPs can induce oxidative stress in vitro model [16]. We first assessed whether NPs induced oxidative stress in cardiomyocytes. ROS levels were examined by laser confocal microscopy, which revealed a significant increase in ROS level (Fig. 8A). In addition, ROS was also significantly increased in vivo (Fig. 8B).

To test the effect of NPs on mitochondrial function, the oxidative phosphorylation status were analyzed by evaluating the oxygen consumption rate (OCR) after 3 h of nanoplastics treatment. We observed a rapid decrease in baseline OCR, ATP production and proton leakage in a dose-dependent manner (Fig. 8C). However, mitochondrial membrane permeability was unaffected by TMRE staining, indicating that cells still maintained mitochondrial integrity (Fig. 8D). It is suggested that the heart aging induced by NPs may be related to impaired mitochondrial function.

Since mitochondria is a major source of ROS production, ROS may disrupt mitochondrial redox homeostasis [17]. Therefore, we used laser confocal microscopy to detect the production of mitochondrial superoxide using MitoSOX (MitoSOX is a sensitive fluorescent probe specifically targeting the redox reaction of mitochondria). The experimental results revealed that the level of mitochondrial ROS was increased after treatment with NPs (Fig. 8E).

To further demonstrate whether ROS are involved in NPs-induced cellular inflammation/senescence, the ROS scavenger (Trolox) and mitochondria-targeted antioxidant MitoQ were utilized (cells were pretreated with 100 μM Trolox for 1 h; cells were pretreated with 1 μM MitoQfor 1 h) (Fig. 8F). The results showed that MitoQ and Trolox effectively increased cell survival (Fig. 8G), reduced cell senescence (Fig. 8H), and improved the cell cycle (Fig. 8I). In addition, Trolox and MitoQ alleviated cellular senescence and inflammatory factors expression (Fig. 8J, K). These experimental data indicate that ROS mediated the cytotoxicity of NPs.

### Mitochondrial calcium overload induced by nanoplastics leads to oxidative stress and cardiomyocyte senescence

**Fig. 9:**
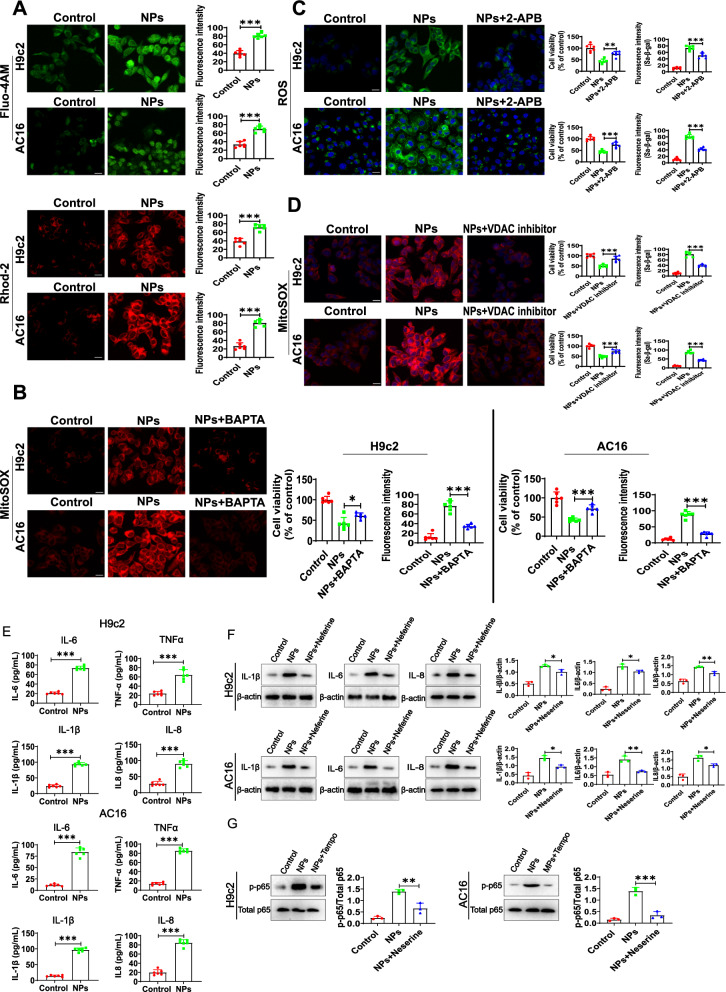
NPs led to mitochondrial calcium overload. **A** Effect of NPs on cytoplasmic and mitochondrial calcium signaling. **B** Effect of BAPTA on ROS, cell viability and cell senescence. **C** Effect of IP3R inhibitor on ROS, cell viability and cell senescence. **D** DIDS (VDAC inhibitor) alleviated cellular senescence. **E** NPs treatment increased inflammation levels. **F** Effect of NF-κB inhibition on inflammation and cell senescence. **G** Effect of ROS inhibition on the NF-κB signaling. An asterisk indicates a significant interference

We further explored the potential molecular mechanisms by which NPs disrupt mitochondrial function. Previous studies have reported that NPs are closely associated with intracellular calcium mobilization [18], and calcium overload in the mitochondrial matrix severely affects mitochondrial function [19]. Therefore, we analyzed the effect of NPs on intracellular calcium mobilization using the cytoplasmic calcium indicator Fluo-4 AM and the mitochondrial-specific calcium indicator Rhod-2 AM. Interestingly, we found that the control group (without nanoplastics treatment) produced weak calcium signals. In contrast, NPs treatment caused a rapid elevation of cytoplasmic/mitochondrial calcium signals (Fig. 9A).

Excessive calcium influx into mitochondria can affect mitochondrial homeostasis and induce oxidative stress. Therefore, we hypothesized that mitochondrial calcium overload leads to mitochondrial dysfunction under NPs treatment, which in turn promotes ROS production and induces the cellular senescence. To this end, we found that NPs-induced mitochondrial ROS levels was decreased when using the calcium chelator BAPTA. Inhibition of ROS increased cell viability and alleviated cellular senescence (Fig. 9B). The accumulation of calcium in mitochondria is dependent on the endoplasmic reticulum, which is the main intracellular calcium storage organelle. Calcium can be released from the endoplasmic reticulum via the inositol 1, 4, and 5-trisphosphate receptor (IP3R). To verify the source of calcium, the IP3R inhibitor (2-APB) was added to cells under nanoplastics treatment, we found that the IP3R inhibitor was able to reduce nanoplastics-induced ROS production, increase cell viability and alleviate cellular senescence (Fig. 9C). Studies have reported that Ca^2+^ released from the endoplasmic reticulum may be transported to the mitochondria via VDAC1, which is located in the outer mitochondrial membrane, and VDAC1 is involved in many biological processes, including calcium homeostasis, cellular senescence, and apoptosis [20]. To demonstrate whether calcium ions enter mitochondria via VDAC, DIDS (VDAC inhibitor) was added to the cells, and results showed that inhibition of VDAC suppressed NPs-induced intracellular ROS production (Fig. 9D, left panel) and cellular senescence (Fig. 9D, right panel). Taken together, our results suggest that NPs can induce ER-mitochondrial calcium overload, which leads to ROS accumulation and cellular senescence.

Next, we asked how the NPs-induced ROS overproduction induces cardiomyocyte senescence. ROS is closely related to inflammation response, which is closely related to senescence [21]. To this end, we analyzed the expression of inflammatory factors in response to NPs treatment, we found an increase in the expression of TNFα, IL-1β, IL6 and IL8 (Fig. 9E). Further experiments found that NF-κB was activated. To determine the relationship of NF-κB with nanoplastics-induced cardiomyocyte senescence, NF-κB inhibitor neferine was used (cells were pre-treated with 5 μM neferine, or NF-κB was knocked down), and results showed that both inflammation and senescence of cells were reduced (Fig. 9F). To identify the relationship between NF-κB and ROS, ROS was inhibited using TEMPO, and NF-κB activation was significantly suppressed (Fig. 9G). These experimental results suggest that mitochondrial ROS may regulate cardiomyocyte senescence by activating NF-κB.

**Fig. 10:**
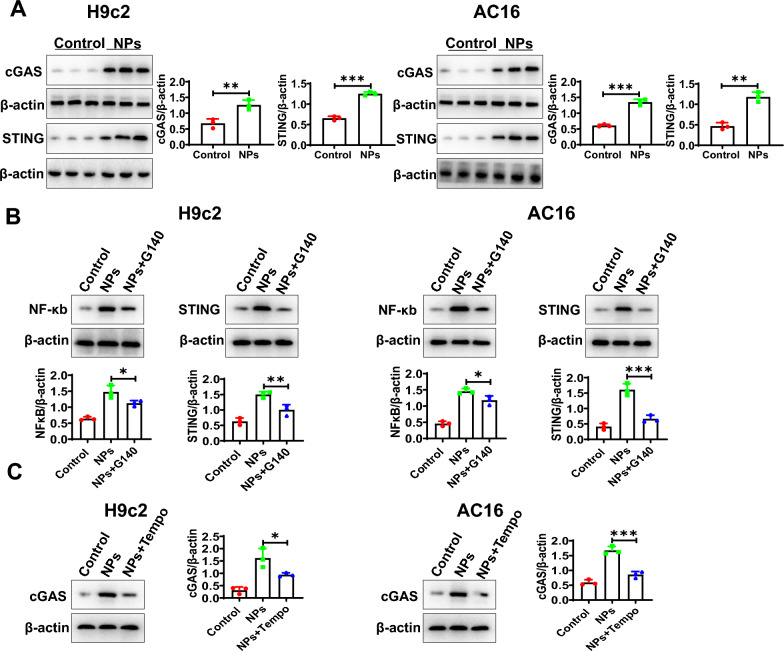
NF-κB activation by cGAS-STING. **A** NPs treatment activated the cGAS-STING signaling pathway. **B** Inhibition of the cGAS-STING signaling pathway alleviated NF-κB activation. **C** Effect of ROS inhibition on the cGAS-STING signaling pathway. An asterisk indicates a significant interference

To determine the mechanism by which mitochondrial ROS activates NF-κB, we analyzed whether the cGAS-STING signaling pathway was activated. Previous study showed that cGAS-STING signaling could activate NF-κB, and microplastics could activate the cGAS-STING signaling pathway [22]. To this end, we conducted the corresponding experiments showing that the cGAS-STING signaling pathway was activated by NPs treatment (Fig. 10A). To demonstrate the relationship between the cGAS-STING signaling pathway and NF-κB under NPs treatment, cGAS-STING signaling pathway inhibitor was used (G140, 5 μM), the experimental data illustrated that the G140 was able to inhibit NF-κB activation (Fig. 10B). To explore the relationship between ROS and cGAS-STING signaling, we found that inhibition of ROS (TEMPO) could block the activation of the cGAS-STING signaling pathway (Fig. 10C). The experimental data suggest that ROS activates NF-κB by cGAS-STING signaling.

### Nanoplastics-induced mitochondrial DNA (mt-DNA) leakage into the cytoplasm

**Fig. 11:**
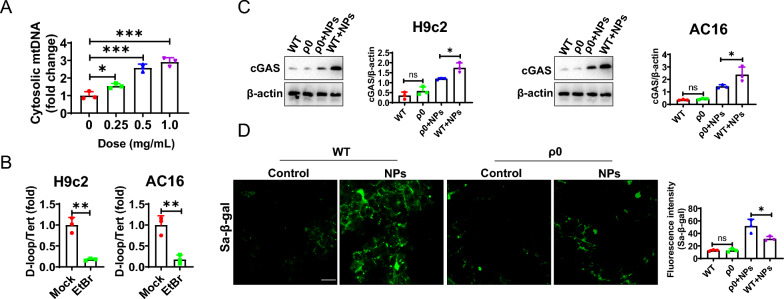
NPs treatment resulted in the leakage of mitochondrial DNA. **A** Nanoplastics treatment resulted in the entry of mitochondrial DNA into the cytoplasm. **B** EtBr treatment removed mitochondrial DNA. **C** The ability of NPs to activate the cGAS-STING signaling pathway was significantly reduced in the ρ0 cell model. **D** NPs-induced senescence and inflammation were attenuated in the ρ0 cells. An asterisk indicates a significant interference

The cGAS-STING signaling pathway induces cellular senescence mainly via sensing DNA in the cytoplasm [23]. Therefore, we hypothesized that NPs treatment may induce the release of mtDNA (mitochondrial DNA) into the cytoplasm as a secondary messenger, which in turn activates the cGAS-STING signaling pathway. To this end, we treated cardiomyocytes with nano-scaled microplastics and then extracted cytoplasmic DNA. We found that nanoplastics treatment induced the accumulation of cytoplasmic mtDNA (Fig. 11A). To determine the function of mt-DNA, ethidium bromide (EtBr) was used to treat cardiomyocytes with the aim of removing mtDNA to generate ρ0 cells [24]. The cells were treated with EtBr for 7 days, resulting in mtDNA loss in more than 80% of cells (referred to as ρ0 cells) (Fig. 11B). We found that nanoplastics-induced cGAS-STING signaling responses were significantly reduced in the ρ0 cell model (Fig. 11C). These data suggest that mitochondrial DNA accumulation in the cytoplasm in the ρ0 cells was significantly reduced in response to nanoplastics stimulation, leading to reduced activation of the cGAS-STING signaling pathway. In addition, we also observed that nanoplastics-induced cardiomyocyte senescence was reduced (Fig. 11D).

### Nanoplastics induce mtDNA release into the cytoplasm via VDAC

**Fig. 12:**
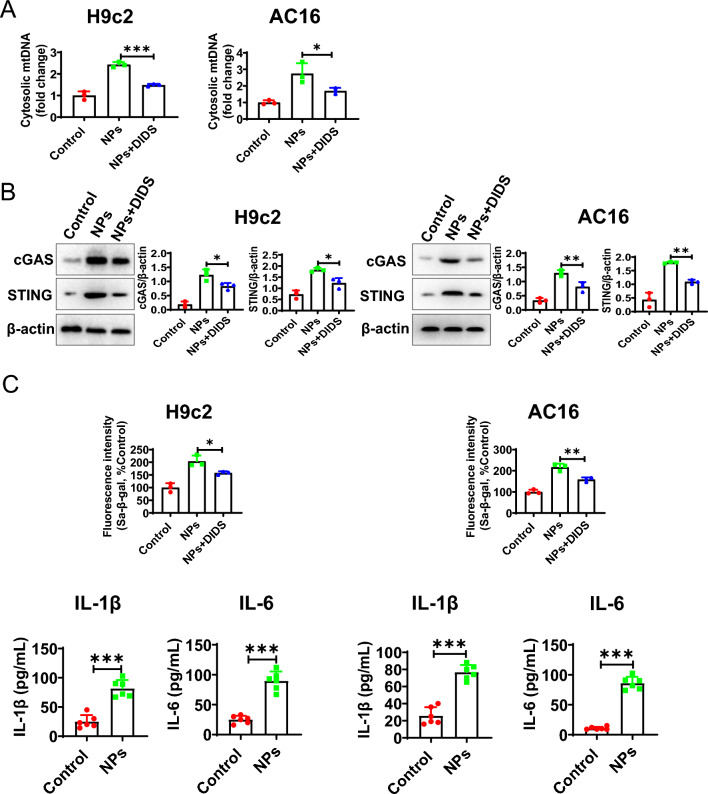
mtDNA leakage into the cytoplasm via VDAC. **A** VDAC inhibitor reduced mtDNA leakage. **B** VDAC inhibitor inhibited the activation of cGAS-STING signaling. **C** VDAC inhibitor alleviated senescence and inflammation. An asterisk indicates a significant interference

In this section, we investigated how mitochondrial mtDNA enters the cytoplasm in the presence of NPs. VDAC was reported to be able to mediate the entry of mtDNA into the cytoplasm [25]. In the above study, we found that VDAC was able to mediate the entry of Ca^2+^ into mitochondria, indicating that VDAC was active. To demonstrate whether mtDNA entry into the cytoplasm was mediated by VDAC, we found that mtDNA in the cytoplasm was significantly reduced under treatment with VDAC inhibitors (DIDS) (Fig. 12A). Meanwhile, the activation of cGAS-STING became weaker (Fig. 12B). In addition, the cellular senescence and inflammation were decreased (Fig. 12C). These results indicate that mtDNA enters the cytoplasm via the VDAC channel.

### Nanoplastics-induced inflammation and senescence were attenuated in cGAS^−/−^ mice

**Fig. 13:**
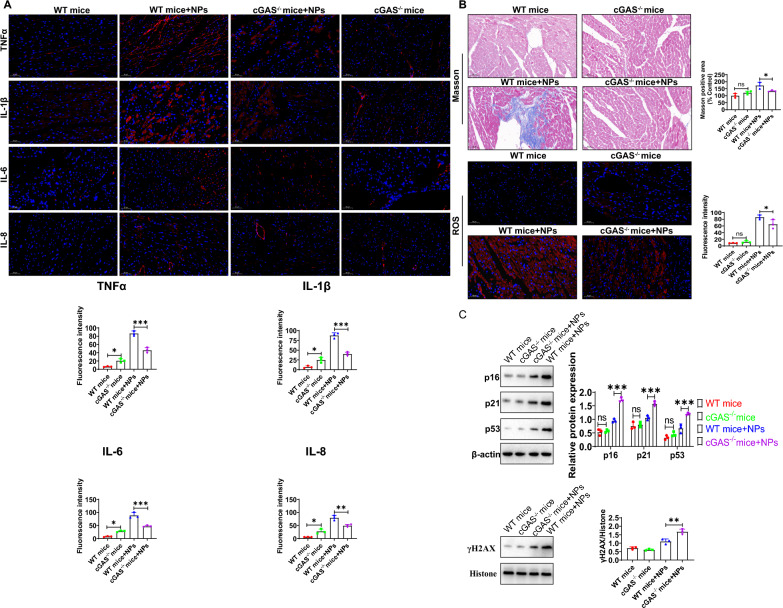
Effects of NPs on inflammation and aging in cGAS knockout mice. **A** NPs-induced inflammation was attenuated in cGAS^−/−^ mice. **B** NPs-induced myocardial fibrosis and oxidative stress were attenuated in cGAS^−/−^ mice. **C** NPs-induced aging was attenuated in cGAS^−/−^ mice. An asterisk indicates a significant interference

In vivo, we sought to explore whether the cGAS-STING signaling pathway is involved in NPs-induced inflammation and aging in cardiac tissue. By immunohistochemical analysis, NPs were able to induce inflammation in cardiac tissue. Compared with WT mice, the cardiac tissue damage caused by nanoplastics was significantly alleviated in cGAS^−/−^ mice, and the expression of TNFα, IL6, IL-1β and IL-8 was significantly reduced (Fig. 13A). Masson staining showed that myocardial fibrosis was significantly alleviated under nanoplastics treatment, and ROS levels were also significantly reduced compared to the control group (Fig. 13B). Furthermore, the expression of aging marker molecules (p16, p21, p53 and γH2AX) were significantly down-regulated in the cGAS^−/−^ mice (Fig. 13C). These evidences indicates that NPs-induced aging is mediated via cGAS-STING signaling pathway (at least partially).

## Discussion

In recent years, nanoplastics have received extensive attention from researchers, and their environmental behavior and toxicological effects have become a research hotspot in the environmental field [26, 27]. Recent studies have shown that drinking water may is a major pathway for nanoplastics to enter the human body and a potential source of health risk [28]. Previous studies have analyzed the health effects of NPs on different organs at various levels [29]. However, as of now, the toxicological effects of NPs on cardiac tissue has not been revealed. For this, we investigated the effects of NPs on cardiac tissues.

In an in vivo model, we found that NPs were distributed in many organs, mainly in the heart and liver and other tissues and organs (Fig. 1). This is consistent with previous reports [29]. In addition, NPs are able to cross the blood‒brain barrier into the brain [30]. However, in the current study, we found that NPs were not able to enter into the brain through the blood‒brain barrier, which may be related to the size of NPs. In addition, we found that NPs are able to cause inflammation, oxidative stress and aging damage in cardiac tissue (Figs. 3 and 4). In addition to the heart, NPs have been reported to cause damage to other organs [31].

In vitro, we further investigated the effect of NPs on cardiomyocytic. Firstly, we investigated whether NPs could transport into cardiomyocytes, and the data showed that NPs could internalize into cells in a time- and dose-dependent manner (Fig. 6). It has also been shown that NPs can be internalized into other cell types [8]. However, the scientific question that remains unresolved is the mechanism by which NPs internalize into cells, and the molecule mediated endocytosis of NPs into cardiomyocytes is still unknown. Nanoplastics caused the cardiomyocytes senescence by characterizing a series of senescence-related marker molecules (Fig. 7). We found that Sa-β-gal expression was increased. H3K27me3 (heterochromatin marker) expression was significantly down-regulated, and γH2AX (DNA damage marker) expression was significantly increased, these observations collectively indicated that NPs caused the cardiomyocytes senescence (Fig. 7).

The oxidative stress is one of the most important factors that cause aging [31]. Cardiomyocytes are rich in mitochondria, and excessive ROS accumulation in the myocardium can lead to myocardial mitochondrial damage [32]. Here, we investigated the molecular mechanisms by which nano-scaled microplastics induce cardiomyocyte senescence. Senescence is highly associated with oxidative stress/inflammation. Therefore, we evaluated the effect of nano-scaled microplastics on oxidative stress. The results showed that NPs caused a significant increase in oxidative stress levels (Fig. 8).

According to the aging-related oxygen radical theory, excessive accumulation of ROS accelerates aging and causes the development of a number of aging-related diseases. Cardiac aging is considered one of the main causes of mortality in the elderly population [33]. The aging of the heart occurs with age and is accompanied by four main changes at the functional, structural, cellular, and molecular levels of the heart. At the molecular level, abnormal mitochondrial function is now recognized as an important factor contributing to cardiac aging [33]. Mitochondria play an important role in cardiomyocyte function and are particularly important in the heart due to the high energy demand of the myocardium [32]. In the current study, we found that excessive accumulation of ROS leads to the release of mtDNA from mitochondria into cytoplasm, which in turn activates the cGAS-STING signaling pathway, thus leading to the cardiomyocyte senescence. Further experiments showed that nano-scaled microplastics may lead to ROS production by inducing calcium overload. However, we cannot exclude the possibility that NPs contribute to senescence partially through other pathways.

Aging is a progressive process that is generally considered to be often accompanied by a time-dependent decline in cellular function and physiological integrity. The prevalence of cardiovascular-related diseases also increases significantly with the progression of aging [34, 35]. Thus, aging is oneof the challenging issues facing the world today. In the current work, we found that NPs acceleratethe aging of cardiomyocytes and tissues. This lays a research foundation for further studying the toxicological effects of NPs.

## Materials and methods

### Reagents and antibodies

P16/INK4a/CDKN2A antibody and p21/CIP1/CDKN1A antibody were purchased from Abcam (UK). Protein lysate RIPA, DMEM, fetal bovine serum (FBS) and protein markers were purchased from Thermo-Fisher. The BCA protein quantification kit, SOD assay kit, MDA assay kit and DMSO were purchased from Solar-bio life science company (Beijing, China). A protein blotting luminescent solution kit was purchased from Solarbio Company (Beijing, China). Fluorescence-labeled nano-scaled microplastic were purchased from Tianjin Bessler Company (Tianjin, China). Nanoplastics were characterized by scanning electron microscopy (Additional file 1: Fig. S1). Trypsin, penicillin solution, Western blot and IP cell lysis solution were purchased from Biyotime Biotechnology Co (Shanghai, China). The Cell Event Senescence Green Detection Kit (C10850) was purchased from invitrogen Company (Shanghai, China). The ELISA Kits (PI305, human IL-1β; PI330, human IL-6; PT518, human TNF-α; PI301, mouse IL-1β; PI326, mouse IL-6; PT512, mouse TNF-α) were purchased from Beyotime (Shanghai, China). Anti-γ-H2AX antibody and anti-β-actin was purchased from Abclonal company (China).

### Cell culture

The H9c2 cardiomyocyte cell line was purchased from ATCC, and the human cardiomyocyte AC16 cell line was from the Cell Bank of Chinese Academy of Sciences. AC16 cells were cultured in DMEM/F-12 medium containing 12% FBS. H9c2 cells were cultured in high sugar DMEM supplemented with 10% FBS.

### Kinetics of cellular nanoplastics internalization

Cardiomyocytes were inoculated onto coverslips and incubated for 10 h. After the cells were washed, fluorescently-coupled nanoplastics (0.1–1 mg/mL) were added to the cell medium and incubated for 0–90 min. The cells were washed three times and fixed with 4% paraformaldehyde for 20 min at room temperature. After three washes with PBS, DAPI was used to stain cell nuclei. Cell samples were examined by an laser confocal microscope (Olympus FV3000).

### Sa-β-Gal staining

The cell medium in the six-well plate was discarded. After three washes with PBS, 1 mL of Sa-β-galactosidase staining fixative was added and incubated at room temperature for 15 min. The cells were washed three times (3 min each) using PBS, and 1 mL of Sa-β-gal staining working solution was added to each well of the six-well plate. The plates were sealed with sealing film and incubated overnight in a CO_2_-free incubator at 37 °C. The next day, the samples were placed under an inverted light microscope for observation.

### Analysis of Sa-β-gal activity by CellEvent™ Senescence Green Detection Kit

SA-β-gal activity was tested using the CellEvent™Senescence Green Detection Kit (Thermo Fisher Scientific, Waltham, USA) according to the manufacturer’s protocol. In brief, the cardiomyocytes were cultured on coverslips in a 6-well cell culture plate for 24 h. The cells were then fixed with 4% PFA for 0.5 h. After rinsing three times with PBS, the cells were treated with Sa-β-gal staining solution under CO_2_-free conditions. The cells were then treated with Hoechst 33342 (2 μg/mL) to stain cell nuclei for 10 min. Finally, the cell samples were checked by an FV3000 laser scanning confocal microscope (Olympus).

### ROS and MDA (malonaldehyde) detection

DCFH-DA was diluted with DMEM medium, and the final working solution concentration was adjusted to 10 µmol/L. The cells was washed three times using PBS, and nanoplastics were added and incubated for 12 h. After washing, DCFH-DA was added and incubated in the incubator at 37 °C for 30 min. After incubation, the DCFH-DA probe working solution was discarded, and DMEM medium was used to wash the cells three times before the cell samples were examined using a laser confocal microscope (Olympus, Japan, FV3000).

MDA (malonaldehyde) level was detected using MDA assay kit according to the manufacturer's instructions (Cat.no. S0131M, Beyotime biotechnology company).

### Lipofectamine 3000 transfection

Cells were first inoculated into 6-well plates. When the cells reached 60%-70% confluence, the old medium was discarded, fresh medium was added and incubated in a cell incubator for 1 h before starting cell transfection. Five microliters of P3000™ reagent was added. The plasmid-containing mixture was mixed with Lipofectamine 3000 reagent and incubated at room temperature for 5 min. The mixture was added equally to 6-well plates and incubated in a cell incubator with 5% CO_2_ at 37 °C. The transfection efficiency was determined by western-blotting.

### Experimental animal administration

All experimental animal manipulations were approved by the Experimental Ethics Committee of Guangzhou Medical University. Prior to the start of the experiments, mice were acclimated by being placed in a 12 h light/dark cycle and at 55% ± 10% relative humidity at 22 ± 2 °C for one week. Mice were randomly divided into 4 groups of 6 mice each: control group, the low-dose (3 mg/kg), medium-dose (6 mg/kg), and high-dose (10 mg/kg) groups (The current choice of nanoplastics exposure dose was based on previous literature [11, 12]). The experimental animals were administered with nanoplastics by oral gavage at 3 mg, 6 mg and 10 mg/kg twice per week for 8 weeks. The mice in the control group were administered with sterile water. After the experiment, the samples were fixed in 10% formalin for further histological analysis.

### Cardiac ultrasound examination

Mice were anesthetized by inhalation of isoflurane, and cardiac Doppler ultrasound was performed by using a Visual Sonic Vevo 2100 (17.5-MHz probe). The mice were also closely monitored for heart rate and electrocardiogram to observe their vital signs and to avoid death due to anesthesia overload. Ejection fraction (EF) and fractional shortening (FS) are commonly used indicators to reflect ventricular systolic function (the physiological range of EF: 55–65%; the physiological range of FS: 25–35%). Ejection fraction (EF) and fractional shortening (FS) were calculated by Simpson’s method as described in [36].

### Extraction of myocardial tissue proteins

The basic procedure for extraction of cardiac tissue proteins was as follows. Frozen heart tissue was quickly removed from the − 80 °C freezer and placed on ice. Approximately 50 mg of each sample was extracted. Samples were washed three times using PBS and placed in 1.5 mL EP tubes. One milliliter of lysis solution was added and placed on ice. Tissues were cut using scissors and transferred to a 1 mL homogenizer for homogenization. The protein suspension in the homogenizer was transferred to an EP tube and fully lysed for 30–40 min. Samples were centrifuged at 12,000 rpm for 20 min at 4 °C. The supernatant was collected and transferred to a freshly precooled EP tube. Protein concentrations were determined using the BCA method according to the kit instructions.

### Western blot

Protein samples from cells or tissues were subjected to SDS‒PAGE and transferred to PVDF membranes. BSA (5%) was then added to seal the PVDF membranes for 2 h at RT. The PVDF membranes were washed three times using TBST (3 min each time), diluted primary antibody was added and incubated at 4 °C for 16 h. After the PVDF membranes were washed, horseradish peroxidase-labeled secondary antibody working solution was added and incubated at 37 °C for 120 min. The secondary antibody was discarded, and the membranes were washed three times using TBST (5 min/wash). ECL (enhanced chemiluminescence) was added to detect immune protein bands. Blots were imaged with the Bio-rad Gel Doc XR^+^ system (Bio-Rad). The band intensities were measured using Image J software (version 1.48, National Institutes of Health, Bethesda, MD, USA) and normalized to those of β-actin (loading control).

### Measurement of total mtDNA

After nanoplastics treatment, the cells were collected. The cells were then lysed. Total DNA was isolated using the commercial kit from Abcam (UK). The samples were centrifuged at 12,000×*g* for 5 min, and then the supernatant was transferred to a new 1.5 ml EP tube. Mitochondrial DNA was quantified by qPCR using primers specific for the mitochondrial D-loop region. The content of D-loop was normalized with the content of nuclear DNA (telomerasereverse transcriptase,Tert).

### H&E staining

The basic H&E staining procedure was as follows: paraffin sections were dewaxed in water. The sections were treated with xylene I for 6 min and transferred to xylene II for 6 min. Samples were transferred to anhydrous ethanol I for 6 min, anhydrous ethanol II for 6 min, 95% ethanol for 3 min, 80% ethanol for 3 min, and 75% ethanol for 3 min. After washing, samples were stained with hematoxylin for 5 min. After washing, the samples were stained with hematoxylin for 5 min. After rinsing with tap water, the samples were stained with 0.5% eosin for 2 min and then rinsed 3–5 times. After dehydration and transparency, samples were sealed with neutral gum and observed and imaged under a microscope.

### Masson staining

Samples were stained using Weigert’s iron hematoxylin for 5 min after washing with tap water. Samples were treated with 1% hydrochloric acid for 20 s. After rinsing with tap water for 5 min, Masson staining solution was added dropwise to the slide for 8 min. After washing, samples were immerged with 2% glacial acetic acid solution. The samples were then treated with 1% phosphomolybdic acid solution for 5 min. Samples were stained with aniline blue for for 5 min. After washing, samples were infiltrated in 0.2% glacial acetic acid solution for 1 min. After dehydration, samples were sealed with neutral gum to observe under the microscope. Quantitative analysis of Masson's trichrome staining was done using ImageJ software (version 1.48, National Institutes of Health). The stained images were opened with ImageJ software, the positive area (blue areas) of collagen fibers were selected by adjusting “colour threshold”. The positive area was calculated using Image J.

### Immunofluorescence staining

The sections were added dropwise with 0.5% Triton at RT for 0.5 h. Samples were washed using PBS. The blocking solution was added for 1 h. After washing, the primary antibody working solution was added and incubated overnight at 4 °C. The sections was washed 3 times (5 min each time), the corresponding secondary antibody was added and incubated at room temperature for 2 h. After washing (5 min each time), the corresponding secondary antibody was added for 120 min. The samples were washed three times with TBST (5 min each time). DAPI was added. After incubation for 15 min, the samples were analyzed under the laser scanning confocal microscopy equipped with the 20× and 60× oil lens (Olympus, FV3000, Japan). Fluorescent images were captured by the Olympus FV3000 FluoView software. Fluorescence images were analyzed using ROI manager module of Fluoview software (Olympus, FV3000).

### Flow cytometry

After the cells were treated with nanoplastics, the cells were digested using EDTA-containing trypsin for 3 min in a 37 °C incubator. After the cells were rounded, 750 µL of serum-containing medium was used to terminate the digestion. Cells were collected by centrifugation. The corresponding staining solution was added and incubated at room temperature for 0.5 h. After centrifugation at 1000 rpm for 10 min, cell precipitates were washed three times using PBS. Then, 200 µL of PBS was added, and the cells were resuspended. Cell samples were assayed using a Flow cytometer (BD).

### ELISA

The levels of the inflammatory factors were detected using ELISA kits according to the manufacturer’s instructions.

### In situ detection of reactive oxygen species (ROS) in cardiac tissues

In the current study, we used ROS in situ assay kits to analyze the ROS levels. The fluorescent probe DHE (DHE-ROS Assay Kit, China) was used to detect ROS levels, and the procedure was performed according to the kit’s instructions.

### Statistical analysis

Experimental results are expressed as the mean values ± standard deviations (SD). Student's t test was used to compare two groups, and ANOVA was used for comparisons between multiple groups. Asterisk (*) indicates a significant difference (p < 0.05); different letters within column indicate significant difference (p < 0.05).

## Conclusions

In summary, we found that nanoplastics exposure caused the senescence of cardiomyocytes. This study lays the research foundation for further exploring the toxicological effects of nanoplastics exposure on cardiac tissues.

### Supplementary Information

**Additional file 1.** Characterization of nanoplastics by SEM.

## Data Availability

The data generated in this work is available from the corresponding author on reasonable request.
