# Supplementary figures and images for: Nanoplastics causes heart aging/myocardial cell senescence through the Ca2+/mtDNA/cGAS-STING signaling cascade
Source: J Nanobiotechnology. 2024 Mar 6;22:96. doi: 10.1186/s12951-024-02375-x (PMC10918962; doi:10.1186/s12951-024-02375-x)

## Slide 1
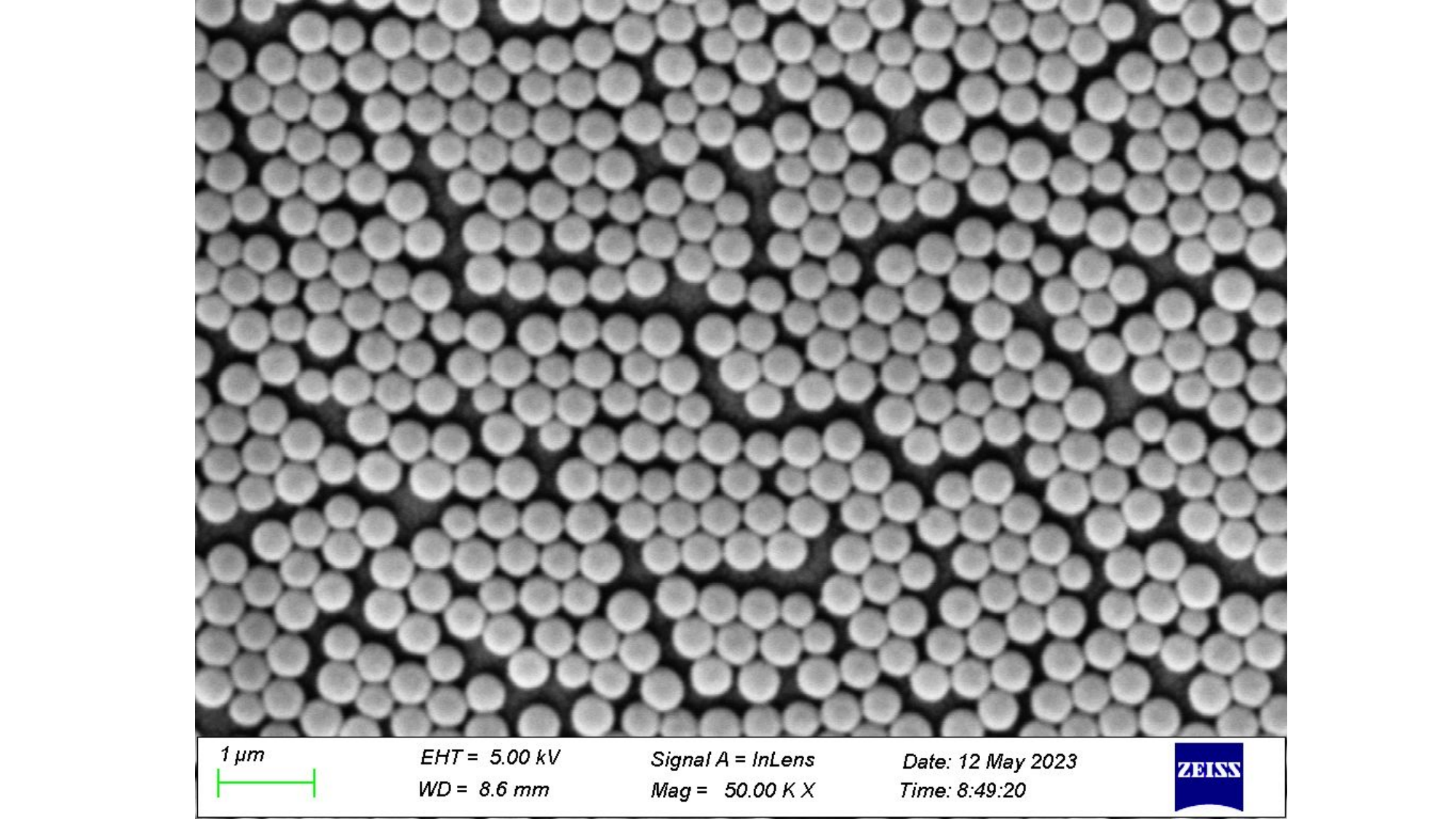

## Slide 2
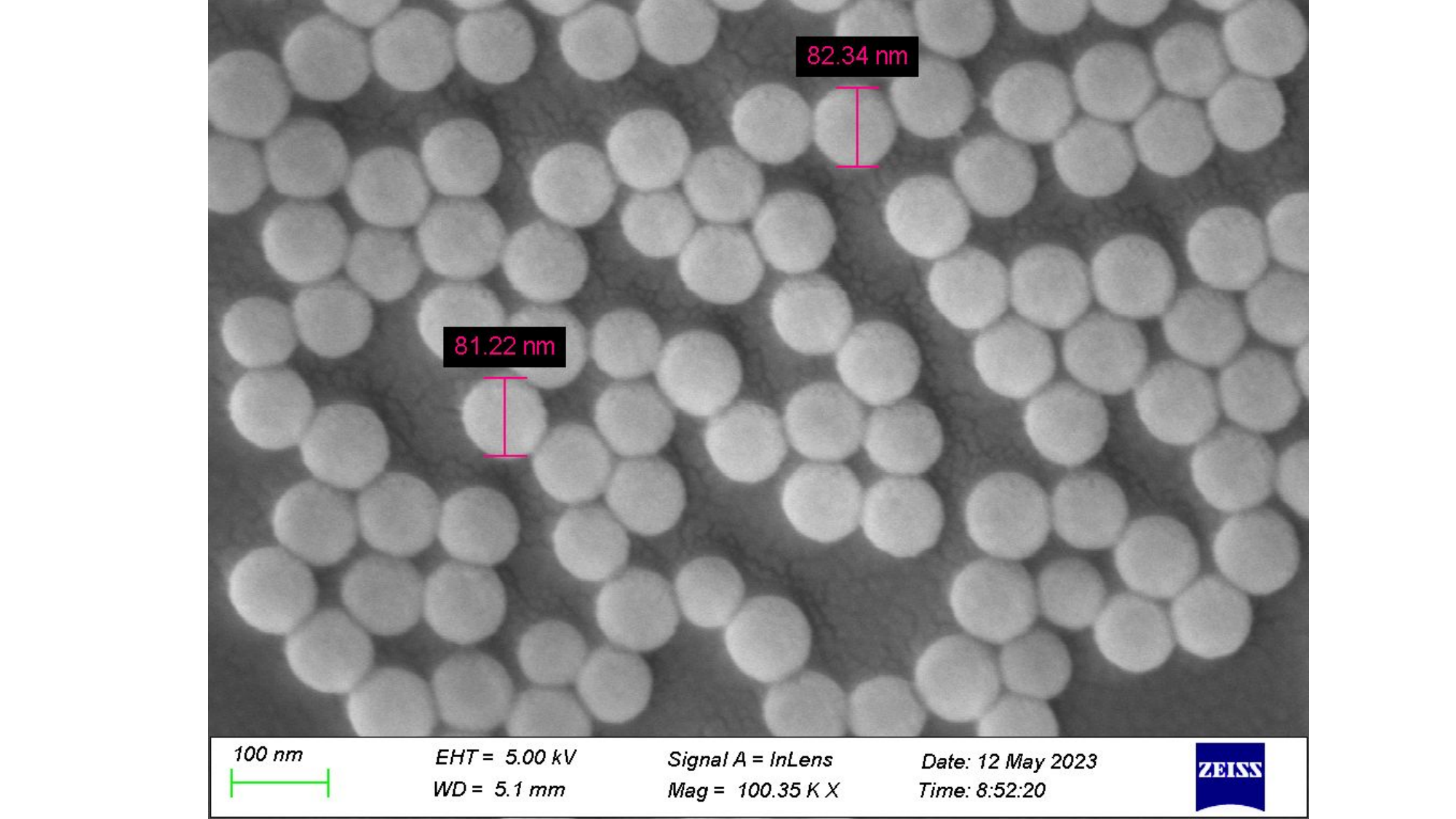

## Slide 3
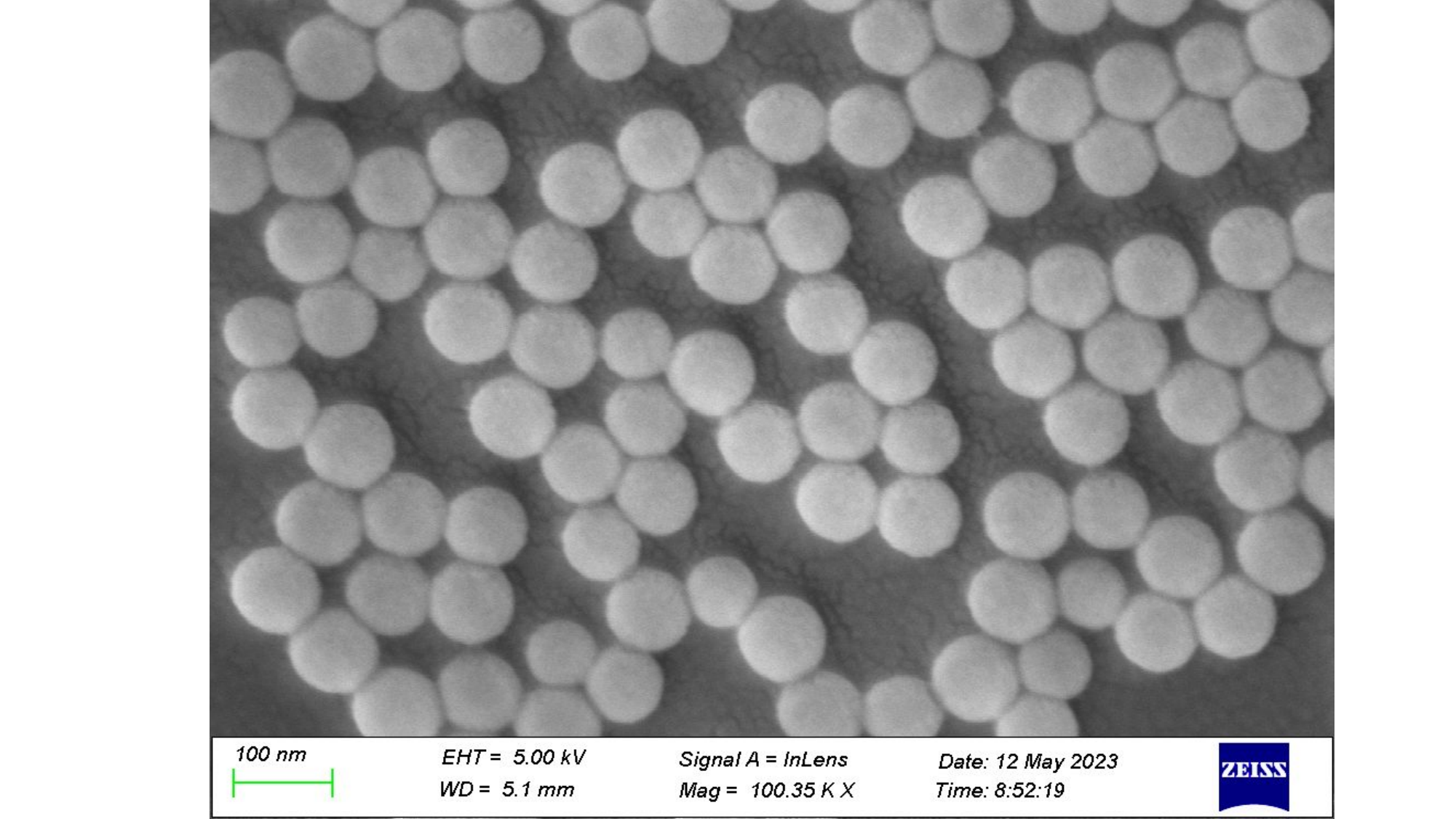

Supplement: Supplementary file 1 — Additional file 1. Characterization of nanoplastics by SEM. [file 12951_2024_2375_MOESM1_ESM.pptx]
